# Supplementary material for: Histological evaluation of the distribution of systemic AA-amyloidosis in nine domestic shorthair cats
Source: PLoS One. 2023 Nov 2;18(11):e0293892. doi: 10.1371/journal.pone.0293892 (PMC10621960; doi:10.1371/journal.pone.0293892)
Supplement: S1 Fig — Amyloidogenic amyloid A aggregates were stained with anti-SAA (a, in red) and ThioflavinS dye (b, green). A merge image was created to visualize co-localization (Merge, c, yellow). Nuclei were stained using DAPI and were coloured blue. (DOCX) [file pone.0293892.s001.docx]

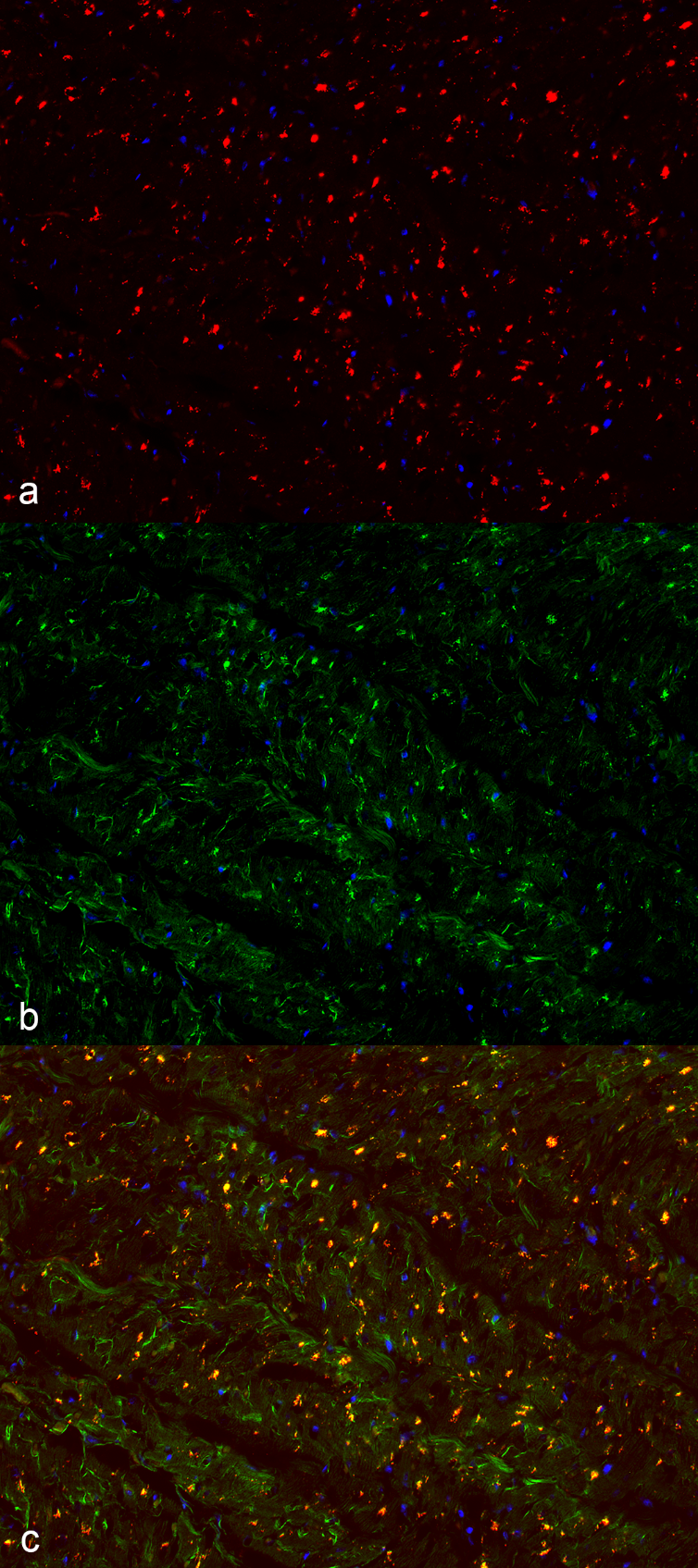


Supplemental figure S1. Immunofluorescent staining AA-amyloid in the heart of a cat. Amyloidogenic amyloid A aggregates were stained with anti-SAA (a, in red) and ThioflavinS dye (b, green). A merge image was created to visualize co-localization (Merge, c, yellow). Nuclei were stained using DAPI and were coloured blue.
