# Supplementary material for: Histological evaluation of the distribution of systemic AA-amyloidosis in nine domestic shorthair cats
Source: PLoS One. 2023 Nov 2;18(11):e0293892. doi: 10.1371/journal.pone.0293892 (PMC10621960; doi:10.1371/journal.pone.0293892)
Supplement: S1 Table — (DOCX) [file pone.0293892.s003.docx]

| **Organs** | **Lesions** | **Amyloid score 1** | **Amyloid score 2** |
| --- | --- | --- | --- |
| Tongue | 1 ulcerative glossitis |  | x |
|  | 1 eosinophilic granuloma |  | x |
| Small intestine | 5 chronic mild/moderate enteritis |  | xxxxx |
| Large intestine | 4 chronic mild/moderate enteritis | x | xxx |
|  | 1 chronic mild enteritis and fibrinous peritonitis |  | x |
| Liver | 1 focal biliary cyst |  | x |
|  | 1 fibrinous-hemorrhagic perihepatitis, suppurative cholangiohepatitis and polycystic disease |  | x |
|  | single cell necrosis associated with hepatocellular disarchitecture and pigment accumulation |  | x |
| Gallbladder | 1 diffuse chronic mild cholecystitis |  | x |
| Pancreas | 1 nodular mild hyperplasia |  | x |
|  | 1 focal mild pancreatitis |  | x |
|  | 1 multifocal mild pancreatitis associated with multifocal atrophy and diffuse hyperplasia |  | x |
| Lung | 1 multifocal interstitial chronic moderate pneumonia associated with atelectasia and emphysema | x |  |
|  | 1 moderate atelectasis with mild oedema |  | x |
| Kidney | 7 interstitial chronic nephritis | x | xxxxxx |
| Urinary bladder | 1 chronic mild cystitis | x |  |
| Adrenal gland | 4 diffuse hyperplasia | x | xxx |
| Spleen | 2 white and red pulp depletion |  | xx |
|  | 1 mild/moderate follicular hyperplasia |  | x |
|  | 1 multifocal leukocytoclastic mild splenitis |  | x |
| Lymph node | 1 reactive lymphadenopathies | x |  |
|  | 1 fibrinous chronic lymphadenitis |  | x |

Supplemental table S3. Summary of the detected lesions in AA-amyloid positive organs and of amyloid score.
