# Supplementary material for: Histological evaluation of the distribution of systemic AA-amyloidosis in nine domestic shorthair cats
Source: PLoS One. 2023 Nov 2;18(11):e0293892. doi: 10.1371/journal.pone.0293892 (PMC10621960; doi:10.1371/journal.pone.0293892)
Supplement: Attachment — Submitted filename: peer review.pdf [file pone.0293892.s004.pdf]

Title of paper:

Histological evaluation of the distribution of systemic AA-amyloidosis in nine domestic shorthair cats

The abstract:

The abstract summarizes the obtained findings, but the histopathological findings need more focusing.

Introduction:

The objective of this study can be written in a better way and more interesting.

The Authers not highlighting the lack of information about the histopathological findings that could be the objective of this study.

In general, the introduction has sufficient information about the nature of amyloidosis and its prevalence.

Material and Methods:

Materials and methods were written subtitled in a good way but why not talk about precautions taken during collecting testes samples?

Consider providing more details on specific staining techniques, reagents and equipment used.

Results:

The results are presented clearly and concisely, with appropriate tables and figures and statistical analysis.

It will be more beneficial to include additional representative images of histopathological findings to enhance the visual understanding of the results.

Discussions:

The discussion provides a comprehensive interpretation of the result in the context of existing knowledge.

Conclusion:

The conclusion succinctly summarizes the main findings and their implications.

It would be helpful to include suggestions for future research directions.

References:

The references are recent and up to date, adding to the currency of the paper.

The references are highly relevant to the topic matter being discussed.

The author appropriately draws connections between the references and their research.

Writing style:

The writing style is clear and concise, making it easy to follow the author's idea.

- Some parts could benefit from additional organization for improved readability.

Additional Comments:

The study represents a valuable contribution to the field of hitopathology and provides novel insights into amyloidosis in cats.
